# A Review of Pavement Damping Characteristics for Mitigating Tire-Pavement Noise: Material Composition and Underlying Mechanisms

**DOI:** 10.3390/ma19030476

**Published:** 2026-01-24

**Authors:** Maoyi Liu, Wei Duan, Ruikun Dong, Mutahar Al-Ammari

**Affiliations:** 1 School of Civil Engineering, Chongqing University, No. 83 Shabei Street, Chongqing 400045, China; liumaoyi@163.com (M.L.); weiweid200@163.com (W.D.); l2400098@stu.cqu.edu.cn (M.A.-A.); 2 Chongqing City Investment Infrastructure Construction Co., Ltd., Chongqing 400015, China; 3 State Key Laboratory of Safety and Resilience of Civil Engineering in Mountain Area, Chongqing 400045, China

**Keywords:** tire-pavement interaction noise, damping materials, damping properties, asphalt binders, polymers

## Abstract

The mitigation of traffic noise is essential for the development of sustainable and livable urban environments, a goal that is directly contingent on addressing tire-pavement interaction noise (TPIN) as the dominant acoustic pollutant at medium to high vehicle speeds. This comprehensive review addresses a critical gap in the literature by systematically analyzing the damping properties of pavement systems through a unified, multi-scale framework—from the molecular-scale viscoelasticity of asphalt binders to the composite performance of asphalt mixtures. The analysis begins by synthesizing state-of-the-art testing and characterization methodologies, which establish a clear connection between macroscopic damping performance and the underlying viscoelastic mechanisms coupled with the microscopic morphology of the binders. Subsequently, the review critically assesses the influence of critical factors, such as polymer modifiers including rubber and Styrene-Butadiene-Styrene (SBS), temperature, and loading frequency. This examination elucidates how these variables govern molecular mobility and relaxation processes to ultimately determine damping efficacy. A central and synthesizing conclusion emphasizes the paramount importance of the asphalt binder’s properties, which serve as the primary determinant of the composite mixture’s overall acoustic performance. By delineating this structure-property-performance relationship across different scales, the review consolidates a foundational scientific framework to guide the rational design and informed material selection for next-generation asphalt pavements. The insights presented not only advance the fundamental understanding of damping mechanisms in pavement materials but also provide actionable strategies for creating quieter and more sustainable transportation infrastructures.

## 1. Introduction

The rapid pace of urbanization accelerates the development of traffic, providing more convenient transportation and thereby fostering economic prosperity. However, it also leads to a significant increase in traffic noise, which impacts tangibly on people’s lives [1,2,3,4]. Donavan [5] conducted a study to quantify the sources of traffic noise, revealing that approximately 78% of the noise was generated from tire-pavement interaction, while vehicle power system and aerodynamic system contributed 12% and 10%, respectively. Notably, power system noise predominates at low speeds in the case of cars. However, once the speed surpasses the critical threshold of 50 km/h, tire-pavement noise becomes the main source of road traffic noise [6]. Additionally, advancements in the automobile industry promote the development of quieter exhaust systems and engines, as well as more efficient aerodynamic systems. Similarly, the extensive models on the dynamic characteristics of tires drive the development of low-noise tires for reducing TPIN at the source [7,8,9,10]. Therefore, reducing TPIN is of great significance for the broader endeavor to mitigate traffic noise in the field of pavement materials.

While the influence of macroscopic parameters like texture and porosity on TPIN is well-documented in previous reviews [11,12,13], a critical disconnect persists in the literature. Existing studies often treat the damping behavior of asphalt pavements as a bulk, empirical property, without tracing its fundamental origins to the molecular viscoelasticity of the asphalt binder and its modified nanostructures. Consequently, there is a lack of a consolidated scientific framework that integrates understanding across scales—from the molecular mobility and relaxation processes governed by modifiers like SBS and crumb rubber, up to the composite mixture’s acoustic damping performance. This gap hinders the rational, physics-informed design of advanced asphalt materials where acoustic performance is a primary engineering parameter.

To bridge this critical knowledge gap, the present review formulates a comprehensive, multi-scale framework to consolidate the interrelationship between material structure, viscoelastic properties, and macroscopic acoustic performance. This work is guided by three principal objectives: first, to systematically synthesize advanced characterization methodologies that link macroscopic damping performance to underlying microscopic morphology and viscoelastic mechanisms; second, to critically evaluate the influence of governing factors—such as polymer modification, temperature, and loading frequency—on molecular mobility and their consequent role in determining damping efficacy; and third, to delineate a definitive structure-property-performance relationship that establishes the asphalt binder as the principal determinant of a pavement’s acoustic signature. The scope is deliberately focused on the damping characteristics of asphalt materials, with particular emphasis on polymer-modified binders and their resultant composite mixtures, thereby providing a foundational scientific basis for the informed design and material selection of next-generation quiet pavements.

## 2. Methodology

This review constructs a scientific framework for the design of quiet asphalt pavements by adopting a systematic, multi-scale analytical approach. The methodology was rigorously designed to ensure a comprehensive and unbiased synthesis of the literature, proceeding through three defined stages: literature collection, critical selection, and analytical synthesis. A systematic search of the Scopus and Web of Science databases was conducted, encompassing publications from January 2000 to March 2024. The search strategy employed key terms related to tire-pavement noise, damping, and asphalt materials, such as (tire-pavement noise, damping, and polymer-modified asphalt). The retrieved literature was subsequently screened against predefined inclusion criteria, prioritizing primary research that explicitly connects the viscoelastic and damping properties of asphalt materials to their acoustic performance.

**Figure 1:**
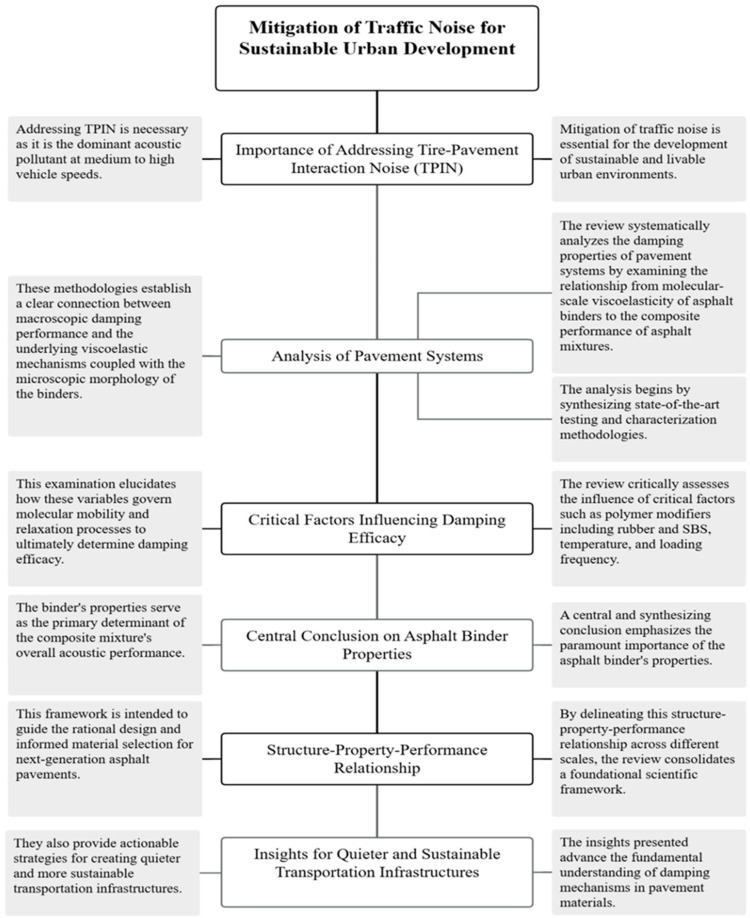
Structure-Property-Performance Workflow for Mitigating Tire-Pavement Interaction Noise (TPIN).

The ensuing analysis, illustrated in Figure 1, is structured around a critical structure-property-performance relationship, tracing mechanistic pathways from the molecular viscoelasticity of the asphalt binder to the macroscopic performance of the composite mixture. Through a thematic analysis that integrates advanced characterization methodologies and key variables—including polymer modification, temperature, and loading frequency—this work establishes the fundamental role of the asphalt binder as the principal determinant of acoustic performance. The consolidation of these insights provides a foundational guide for the rational design of next-generation, sustainable transportation infrastructure.

**Figure 2:**
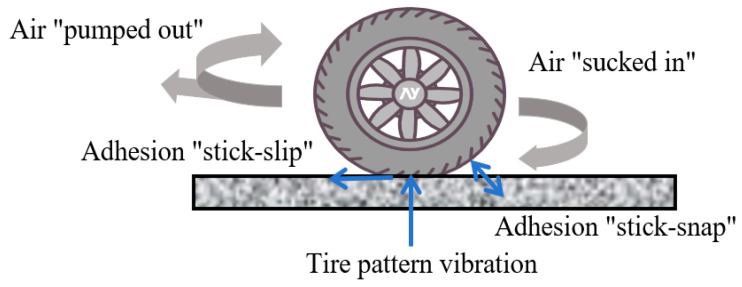
The mechanisms of noise generation in tire-pavement interaction [16].

Tire-pavement interaction noise is defined as the noise emitted from rolling tires due to the interaction between a tire and pavement surface [14,15]. Several noise-generating mechanisms work in conjunction to generate noise when tires roll on the pavement, which are divided into three categories: tire pattern block vibration, adhesion effect and pumping effect, as shown in Figure 2 [16]. Where the noise produced by the contact vibration between tires and pavement is the primary source of TPIN. The adhesion effect is mainly caused by the stick-slip and stick-snap behavior between the tire and pavement. The pumping effect is generated by air movements in the tire pattern cavity and other mechanisms, including air turbulence and pumping, tire groove resonance, air resonance radiation [17]. Therefore, the prevailing theory posits that Tire-Pavement Interaction Noise (TPIN) originates primarily from two key mechanisms: the structural vibration of the tire itself and the aerodynamic pumping of air within the tread-pavement interface [18].

**Figure 3:**
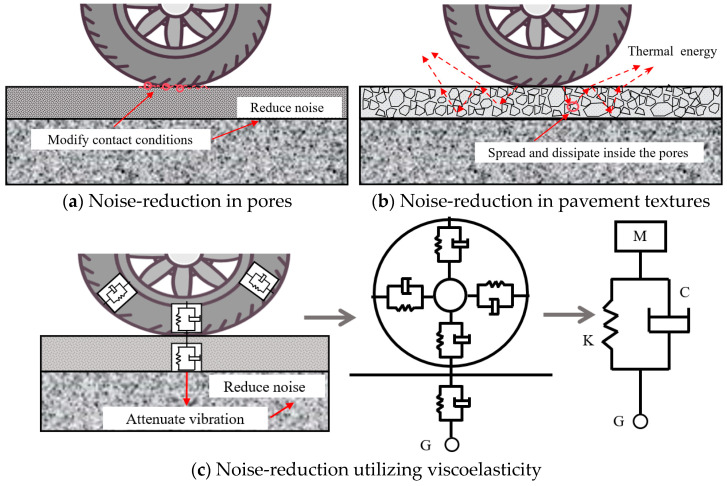
The mechanisms of noise-reduction in pavement [18].

Asphalt pavements are proved to be effective in reducing vibration and noise [19,20]. At present, three mechanisms to reduce TPIN in the pavement field are optimizing the pavement textures, increasing the porosity of mixtures and improving the viscoelasticity of pavement materials, as depicted in Figure 3 [21,22,23]. The spatial morphology and longitudinal depth of pavement textures exhibited the most significant effect on vibration and noise, as declared by Li and Liang [24,25]. The pavement textures affect the volume of the air cavity at tire–pavement contact surface, thereby impacting noise absorption and dissipation [26,27]. Meanwhile, textures modify the contact conditions between tires and pavement, exhibiting significant influence on the TPIN (Figure 3a). The generation and propagation of TPIN are affected by the sound absorption performance of pavement, where the porosity is an essential factor affecting the noise absorption effect of materials [28,29]. Porous asphalt mixtures are characterized by the transmission of sound waves to the gaps in the pavement surface, where they are constantly reflected by colliding and rubbing against the gap wall, converting part of the acoustic energy into thermal energy. Moreover, more pores also imply that the longer propagation of the noise through pavement, resulting in more acoustic energy being lost in the process [30], contributing to noise reduction (Figure 3b). Therefore, the noise-reduction mechanisms of porous mixtures primarily involve the transformation and attenuation of acoustic energy as sound waves propagate through the pores. Noise-reduction utilizing viscoelasticity involves altering the viscoelastic properties of pavement materials to enhance the damping properties. This reduces noise by minimizing the impact vibration between the tire and pavement, with the vibration energy being converted into dissipated thermal energy (Figure 3c) [31,32].

Conventional noise-reduction strategies for pavements, such as optimizing texture and porosity, are often constrained by their transient efficacy and negative impact on durability. In response, this research investigates a more integrated approach by leveraging the inherent damping properties of pavement materials. This method targets the fundamental mechanisms of tire-pavement interaction noise (TPIN) and offers the dual benefit of potentially enhancing pavement performance. Our analysis focuses on the damping behavior and mechanisms of asphalt binders, establishing a theoretical framework to guide the development of next-generation, high-damping materials for quieter and more resilient roadways.

Our analysis consolidates international studies on noise-reducing asphalt, demonstrating how material choices, structure, and surface texture work in concert to mitigate traffic noise and enhance longevity. This synthesis, which directly links acoustic and mechanical performance, answers our central research questions and charts a course for improved pavement design and policy, as detailed in Table 1.

## 3. Damping Behaviors of Materials

Viscoelastic materials present both viscous and elastic properties, determined by the molecular structures [74]. The elastic properties of materials are characterized by the relative slip and torsion of molecular chain segments under external forces, the deformed molecular chains and relative motion between molecules return to their original positions with the removal of external forces. Viscosity properties are featured by overcoming friction and promoting dislocation of intermolecular chains, where some of the vibrational energy is converted into thermal energy. Nevertheless, slip and torsion between the molecular chains of viscoelastic damping materials fail to recover completely, thereby causing the permanent deformation [75,76]. Therefore, viscoelastic materials exhibit both the fluid viscosity the solid elasticity under external forces. Elastic materials store energy and recover their deformation without dissipating energy during deformation. Conversely, viscous materials dissipate energy and their deformation fails to be recovered. Accordingly, viscoelastic materials both store and dissipate part of the energy when deformed by external forces [77,78,79].

**Figure 4:**
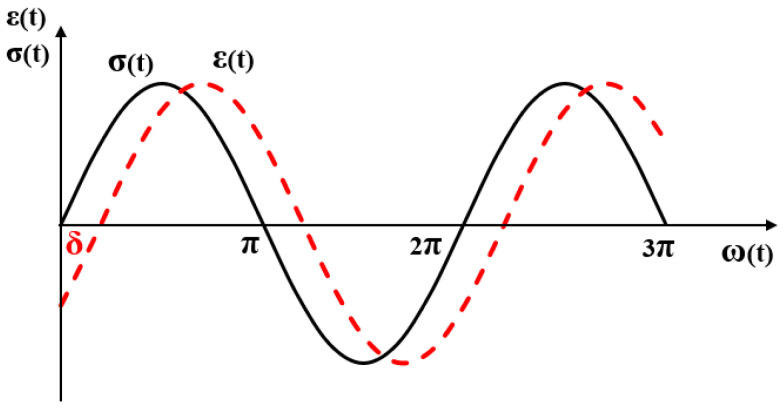
Relationship between stress and strain.

The viscoelasticity of materials is the fundamental cause of their damping properties [80,81]. The process of viscoelastic materials from the macromolecule deformation under external force to equilibrium state, then to the adaptation of the external force is referred to as the relaxation process. The time required for this process is termed relaxation time. The strain of materials lags behind the stress, and the phenomenon is described as the “hysteresis effect”. This leads to the energy loss in the dynamic deformation of materials and performs the damping behaviors [82]. As the viscoelastic materials maintain a stable amplitude and reach equilibrium under alternating loads, both stress and strain vary sinusoidally [83,84,85]. The stress and strain versus time are depicted in Figure 4.

Materials with different properties exhibit various degrees of strain hysteresis response under periodic sinusoidal alternating stress. The strain response of ideal elastic materials is instantaneous and the phase lag between stress and strain δ=0. The hysteresis angle of ideal viscous materials δ=π/2. The hysteresis angle of viscoelastic materials 0 < δ < π/2. The larger δ values indicate that materials are characterized by more hysteresis and higher mechanical loss. In this case, the strain of viscoelastic materials at time t under dynamic load is represented as Equation (1).

(1) $$\varepsilon_{t}=\varepsilon_{0}\left(\sin\;\mathrm{\omega t}\cos\;\delta +\cos\;\mathrm{\omega t}\sin\;\delta \right)$$

From the above equation, the strain of viscoelastic materials consists of two parts. One is elastic strain, manifesting as instantaneous response to stress. The other is viscous strain, existing with a hysteresis δ. The ratio of stress to strain equals the complex modulus of materials, expressed as:

(2) $$\varepsilon_{t}=\varepsilon_{0}\left(\sin\;\mathrm{\omega t}\cos\;\delta +\cos\;\mathrm{\omega t}\sin\;\delta \right)$$

(3) $$G^{\prime }=\frac{\sigma_{0}}{\varepsilon_{0}}\cos\;\delta$$

(4) $$G^{\prime \prime }=\frac{\sigma_{0}}{\varepsilon_{0}}\sin\;\delta$$

where G* represents the complex modulus, G″ represents the loss modulus, G′ represents the storage modulus, δ represents the lag angle.

The storage modulus (G′) is the actual part of complex modulus, representing the energy stored in the elastic deformation of materials. It is usually associated with the stiffness and determines the stiffness or fragility of materials. The loss modulus (G″) is the imaginary part of complex modulus, representing the energy lost in the viscous deformation of materials. The loss modulus is often connected to internal friction and sensitive to various kinds of molecular motion, morphology and structures of molecular [86,87]. Generally, the tangent value of the phase angle δ (also known as the mechanical loss angle) is denoted as loss factor to characterize the damping properties of materials. It is defined as the ratio of the loss modulus to storage modulus [88,89].

**Figure 5:**
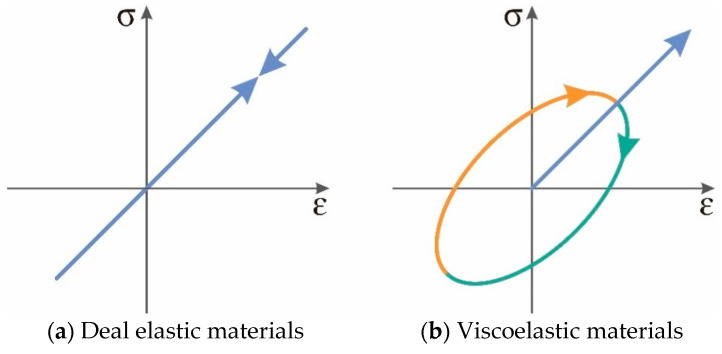
The stress–strain curves of ideal elastic materials and viscoelastic materials.

Damping refers to the dissipation of energy by materials, being achieved by transferring the mechanical vibration energy into thermal energy or other dissipated energy [90,91,92]. Asphalt binders are typically viscoelastic materials and their stress–strain curves differ from that those elastic materials during tension-recovery period. The stress and strain of elastic materials increase or decrease almost simultaneously, resulting in the same or very close phase between them when alternating stress is applied. Therefore, the stress–strain curve of elastic materials is a straight line (Figure 5a). However, the strain of the viscoelastic asphalt binders lags behind the stress under alternating stress, leading to an elliptical hysteresis loop in the stress–strain curve, as depicted in Figure 5b. The hysteresis strain requires overcoming a large damping action, converting the work performed by the external force into dissipated energy, which is the reason that asphalt binders exhibit damping properties.

For the stress–strain curve of viscoelastic materials (Figure 5b), within the first quadrant region, the irregular area formed by the tensile curve with the horizontal axis is the work performed by the external force on materials during the tensile process. The area formed by the retracted curve with the horizontal axis presents the work performed on the outside of materials during the retraction process. The difference in area between the two parts is the work done by the asphalt binders to overcome the resistance of molecular chains, and the lost energy is the vibrational energy absorbed under alternating stresses. Thus, the region enclosed by the elliptical hysteresis line represents the energy dissipated by the asphalt binders during the tire-pavement interaction. Equation (5) is presented below:

(5) $$\begin{array}{ll} \Delta W=\oint \sigma \left(t\right)d\varepsilon \left(t\right) & =\oint \sigma_{0}\sin\left(\omega t\right)d\left[\varepsilon_{0}\sin\left(\omega t\;-\;\delta \right)\right]\\ & =\int_{0}^{\frac{2\pi }{\omega }}\sigma_{0}\varepsilon_{0}\sin(\omega t+\delta )\cos\;\mathrm{\omega t}\mathrm{dt}\\ & =\pi \sigma_{0}\varepsilon_{0}\sin\delta =\pi \varepsilon_{0}^{2}G^{\prime }\tan\;\delta =\pi \varepsilon_{0}^{2}G^{\prime \prime } \end{array}$$

The energy loss capacity of asphalt binders is mainly related to the loss modulus G″ or the tangent value of the phase angle tan δ.

## 4. Evaluation on Damping Properties of Pavement Materials

The imperative for more durable and comfortable transportation infrastructure is a key driver of innovation in pavement engineering. A central research thrust in this domain focuses on enhancing material damping—the capacity to dissipate mechanical energy from traffic-induced vibrations. Compelling evidence, as consolidated in Table 2, identifies the modification of asphalt binders with recycled crumb rubber and specialized polymers as a particularly efficacious strategy. This approach confers a dual performance benefit: a significant mitigation of tire-pavement interaction noise alongside improved resistance to mechanical distress, thereby contributing to an extended service life.

### 4.1. Damping Properties of Asphalt Binders

The viscoelastic nature of asphalt pavement confers a significant performance benefit by actively mitigating traffic-induced noise and vibration, a distinct advantage over rigid Portland cement concrete. This functionality stems from the material’s inherent capacity to dissipate mechanical energy at the tire-pavement interface, effectively serving as an integrated damping mechanism [98]. Given its importance, this energy-dissipating characteristic warrants consideration not as a passive attribute but as an active and fundamental design parameter in pavement engineering, especially for projects in noise-sensitive environments [99]. The underlying physical process is one of internal friction, whereby vibrational energy from loading is converted into heat through the interaction between the asphalt binder and mineral aggregates, thereby attenuating the propagation of acoustic and vibrational waves [100]. To systematically enhance this behavior, polymer modification is extensively employed. Incorporating additives such as styrene-butadiene-styrene (SBS) and crumb rubber (CR) improves the binder’s performance by broadening its range of elastic response across varied service temperatures, which directly augments its damping capacity [101]. The efficacy of these modifiers originates at the molecular scale, where properties like polymer chain flexibility and elastic hysteresis—the lag in molecular response to stress—are the principal determinants of macroscopic damping performance [102]. A critical property of crumb rubber, for instance, is its role in diminishing the asphalt’s thermal susceptibility, thereby ensuring consistent damping efficacy despite seasonal temperature variations [103]. This is accomplished by augmenting the binder’s elastic component and fostering a cross-linked internal microstructure, thereby engineering a more resilient and acoustically optimized composite material.

The addition of a low-damping modifier to SBS-modified asphalt reduces endogenous heat by improving the binder’s elasticity. This improvement is linked to a significant morphological evolution of the SBS phase, transitioning from a state of dispersed, uneven particles to a denser, more homogeneous, and extensively crosslinked network as modifier content increases. At an optimal dosage of 6%, the SBS phase achieves maximum density and uniformity. The resulting synergistic SBS-rubber network enhances the binder’s structural integrity and energy dissipation capacity, explaining the superior damping performance [104].

In recent years, researchers have focused on the damping properties of epoxy asphalt binders. Most of them adopted the damping factor maximum (tan δ_max_), the temperature range (ΔT) for efficient damping (tan δ > 0.3) and the area under the tan δ versus temperature curve (TA) as the evaluation indexes to quantitatively evaluate the damping properties of epoxy asphalt binders [105,106,107]. Experimental results demonstrated that the incorporation of asphalt into an epoxy matrix significantly enhances the composite’s damping parameters. The epoxy asphalt binders exhibited superior damping properties compared to the unmodified epoxy resin, which aligns with previous studies [108,109]. The damping characteristics of epoxy asphalt binders are enhanced through two primary mechanisms related to composition. Firstly, the incorporation of asphalt extends the effective damping interval and elevates the transition temperature (TA) of the epoxy resin matrix. Secondly, as identified by [110], an increase in the epoxy fraction itself contributes to improved damping by shifting the tan δ peak to a lower temperature.

The mechanisms of epoxy asphalt performing enhanced damping properties were further investigated [111,112]. According to quantitative analysis, the incorporation of Sasobit and Waste Cooking Oil (WCO) as warm mix modifiers significantly improved the damping performance of the base epoxy asphalt, a conclusion supported by microstructural evidence from Laser Scanning Confocal Microscopy (LSCM) [96]. This is attributed to the improvement of AR dispersion in the epoxy resin matrix. Pure EAR exhibited that the black discontinuous phase was dispersed in the continuous phase formed by the yellow epoxy crosslinked network. Since a weak interaction between CR and the epoxy matrix, as well as a strong physical cross-linking between CR and asphalt, the dispersed phase refers to AR particles in pure EAR. This implied the two-phase separated structure of EAR, including the phase separation of CR and asphalt, as well as the phase separation between AR and epoxy resin. AR aggregations decreased significantly with the increase in WMA contents. Meanwhile, a more homogeneous phase separation structure was formed in Sasobit-modified EAR. This is attributed to the viscosity-lowering effect of Sasobit on pure EAR, leading to an easier nucleation growth process and consequently to the continued formation of spherical AR particles in the continuous phase of the epoxy resin. Furthermore, a higher WMA content increased the dispersion of asphalt particles within the epoxy resin phase. This aligns with prior findings that the asphalt content primarily influences the mechanical properties of epoxy asphalt by governing the distribution and uniformity of its particulate morphology [113]. Further research confirmed that when the epoxy resin content surpasses 40%, it forms a continuous, three-dimensional crosslinked network [110]. This structure encapsulates the asphalt, thereby enhancing the composite’s capacity to absorb and dissipate vibrational energy.

### 4.2. Damping Properties of Asphalt Mixtures

The acoustic performance of pavement materials has been extensively investigated through the lens of their viscoelastic and damping properties. Initial research creatively established acoustic damping parameters as a key metric, identifying asphalt mixtures as particularly effective for noise reduction [19]. This understanding was refined by a study linking tire-pavement noise to the damping characteristics of asphalt, specifically through the analysis of phase angles [114]. The viscoelastic paradigm was further reinforced by work correlating noise reduction performance with dynamic modulus and phase angle measurements [115]. Deeper mechanistic insight was provided by an analysis of viscoelastic behavior under alternating stresses, which established a definitive relationship between damping properties and the material’s viscosity coefficient [87]. Complementing these approaches, the damping characteristics of CA mortar and asphalt mixtures were quantitatively evaluated using the stress–strain hysteresis area from dynamic creep tests, providing a direct measure of the damping factor [116]. Collectively, these studies underscore the critical role of material damping as a fundamental property governing tire-pavement noise generation.

Furthermore, the incorporation of certain modifiers—including rubber particles, polymers, and fibers—has been shown to enhance the noise-absorption properties of asphalt mixtures. Research indicates that dense-graded pavements with a specific rubber content, such as 3%, demonstrate optimal vibration and noise reduction [117]. The efficacy of this approach is attributed to the increased damping capacity imparted by the rubber particles, with larger particle sizes reportedly leading to greater reductions in vibration and noise [118]. Compared to conventional dense-graded mixtures, rubberized asphalt mixtures exhibit superior acoustic performance, a characteristic explained by the high-viscosity damping effect of the modified binder coupled with the vibration attenuation provided by the rubber itself [119]. This synergistic effect results in a significant optimization of the pavement’s vibration and noise reduction performance, which is further enhanced with increasing rubber asphalt content [120]. The application of waste rubber in porous asphalt mixtures is particularly effective, achieving multi-mechanism noise reduction through high porosity for sound absorption and the material’s inherent damping properties for vibration control [121].

The damping performance of the mixture is also highly dependent on its composition. Lower asphalt contents, which result in a thinner asphalt film coating the aggregate, can inhibit the material’s ability to fully exhibit its damping behavior. The damping properties of an asphalt pavement are derived primarily from the viscoelasticity of the asphalt binder and any added polymers, rather than from the rigid mineral aggregates [122]. This viscoelastic character is influenced by factors such as the binder’s properties, the thickness of the asphalt film, and the interactive forces within the mineral skeleton [98]. Consequently, the damping performance of the composite asphalt mixture and the final pavement is fundamentally inherited from the viscoelastic characteristics of the asphalt binder itself [123].

## 5. Factors Affecting Damping Properties of Pavement Materials

The damping properties of pavement are predominantly governed by the characteristics of the asphalt binder, making it the central element for material evaluation and factor analysis. These viscoelastic damping properties are influenced by multiple parameters, such as molecular structure, crosslinking crystallization, blending system, and environmental conditions. The underlying mechanism connects damping performance to molecular motion, where the material’s composition, alongside external variables like temperature and frequency, is critical in determining the loss factor [124,125,126,127].

### 5.1. Polymer Modifiers

The damping properties of viscoelastic materials are mainly contributed to by polymers, while the damping properties of pavement materials are provided by asphalt and modifiers. In this paper, two polymer modifiers (crumb rubber and SBS) are investigated for their damping mechanisms on the molecular motion scale.

#### 5.1.1. Rubber

Crumb rubber generally consists of complex chemical compositions, including natural rubber (NR), synthetic rubber (SR), butadiene rubber (BR), in addition to additives such as sulfur (S), carbon black (C), and oxides [128]. The molecular structure and morphology of crumb rubber are the fundamental factors dictating its influence on the damping performance of asphalt binders [129,130]. Generally, rubbers with better flexible molecular chains present lower glass transition temperatures, exhibiting poor damping properties. Rubbers with polar side groups, strongly interacting groups, as well as groups with greater spatial site resistance are less flexible, generating more friction from the molecular chain motions and dissipating thermal energy better, thus embodying better damping properties. The rubbers with abundant side groups embody excellent damping properties [131,132].

**Figure 6:**
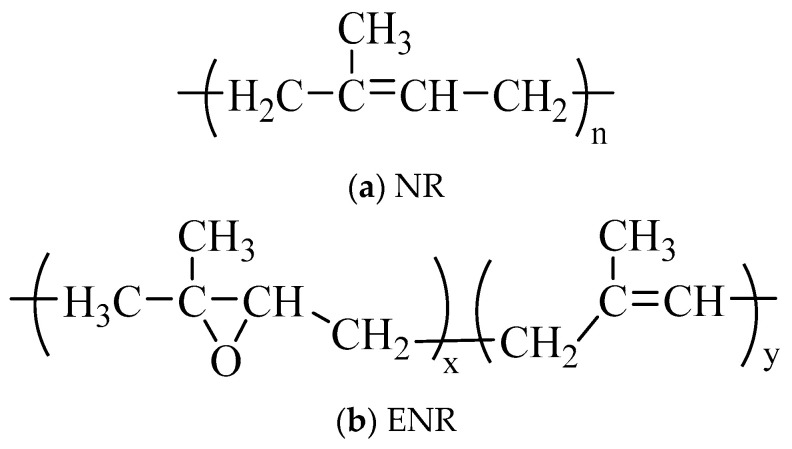
Molecular structure of NR and ENR.

Guided by group contribution molecular theory and the quantitative theory of polymer damping [133], the molecular structure of natural rubber (NR) is intrinsically limited for damping applications, as the double bonds in its monomeric units and the high flexibility of its linear chains result in a narrow damping temperature range. Although polymerization, which introduces methyl side groups, improves damping through enhanced elastic hysteresis, homopolymers like NR seldom provide the high loss factors required across a broad frequency range, necessitating their incorporation into composites to achieve requisite performance [134]. Consequently, the damping properties of NR are frequently enhanced through chemical modification, such as epoxidation to create ENR, which broadens the damping temperature range [135], or through blending with other polymers; for instance, NR/BIIR composites demonstrate that increasing side group complexity widens the effective damping range and increases the loss factor [136]. ENR is particularly effective as it retains NR’s main chain structure and mechanical properties while the introduced epoxy groups increase chain polarity and steric hindrance, thereby amplifying energy dissipation during chain segment motion (see Figure 6. for a molecular structure comparison). A parallel reinforcement mechanism is achieved using nanocomposites, where the incorporation of nanoparticles enhances damping through friction and the sliding of rubber chains against the particle surfaces [137,138].

Styrene-butadiene rubber (SBR) is a synthetic polymer celebrated for its exceptional damping properties. This performance stems from its molecular structure; the bulky benzene rings create a steric hindrance that, combined with the viscous forces of ethylene side groups, restricts intramolecular rotation and friction. This interaction significantly enhances the elastic hysteresis within the material, making SBR a premier choice for damping applications [139]. The utility of SBR as a performance enhancer is well-documented across various fields. For example, research has shown that incorporating SBR into cement paste substantially improves its damping capacity and viscoelasticity [140]. In polymer science, studies on adhesives using a sulfur/accelerator vulcanization system have explored the crosslinking structures of natural rubber (NR) and SBR blends, though findings indicate that some NR/SBR composites can exhibit two distinct tan δ peaks, hinting at potential compatibility issues within the system [141,142,143]. The quest for improved performance has also led to promising composite materials. Investigations into blending SBR with Eucalyptus Ulmoides Gum (EUG) have demonstrated that EUG not only improves the damping properties of the composite but also enhances its overall mechanical characteristics compared to pure SBR [144]. This damping enhancement, particularly at elevated temperatures, has been consistently confirmed in subsequent studies on SBR/EUG composites [145].

Consequently, the presence of SBR and other similar components in crumb rubber is precisely what makes it such an effective modifier for asphalt. By integrating crumb rubber into the asphalt binder, the damping properties of the pavement are significantly enhanced. As vehicle tires roll over the surface, vibrations travel into the pavement structure, where they are absorbed by the modified binder. This energy causes internal displacement and friction within the asphalt matrix, effectively converting and dissipating a substantial amount of vibrational energy and leading to superior noise and vibration reduction.

#### 5.1.2. Molecular Damping Mechanisms of SBS

**Figure 7:**
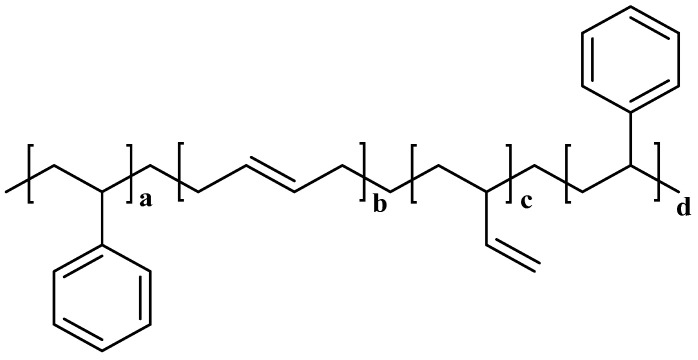
The molecular structure of SBS. a = PS block, b = high trans-1,4 PB, c = high vinyl-1,2 PB, d = high cis-1,4 PB.

Styrene-butadiene-styrene (SBS) is triblock copolymer, the molecular structure is depicted in Figure 7 [146]. There is no chemical bonding between the linear SBS molecules, but rather by van der Waals forces and intermolecular hydrogen bonds [147]. Under the effect of external temperature, the molecular chains slide against each other, giving SBS molecule high damping energy dissipation capacity, good ductility and flexibility. Butadiene in the SBS molecular structure has unsaturated double bonds, which improve the flexibility of the backbone of SBS molecule. According to the group contribution theory, the existence of unsaturated double bonds of butadiene improves the damping properties. In addition, the introduction of benzene rings with large steric hindrance on the side group of SBS molecular structure increases the elastic hysteresis effect of the molecular structure. This provides SBS the enhanced damping properties.

The efficacy of styrene-butadiene-styrene (SBS) triblock copolymer in modifying asphalt is fundamentally governed by the distinct interactions of its polystyrene (PS) and polybutadiene (PB) blocks with the asphalt constituents. Research indicates that intermolecular interactions are stronger between asphalt and the PB blocks compared to the PS blocks [103]. This is attributed to the PB blocks interacting with positively charged groups in asphalt via their π-electrons, while the PS blocks engage with electron-rich groups in asphalt through their aromatic protons [148]. The resulting microstructure is characterized by rigid PS end-blocks aggregating to form physical cross-linking points, thereby establishing a three-dimensional network, while the flexible PB mid-blocks absorb light components from the asphalt (maltenes) and swell, promoting an even dispersion of the SBS particles [149,150]. Within the temperature range bounded by the glass transition temperatures (Tg) of the PB blocks (approximately −90 °C) and the PS blocks (approximately 100 °C), the PB blocks exist in a rubbery state that imparts elasticity and toughness to the binder, whereas the PS blocks are in a glassy state, providing strength and rigidity [151,152]. This synergistic combination, where the hard PS domains reinforce the material and the soft, swollen PB domains enhance flexibility and low-temperature crack resistance, is what establishes SBS as one of the most effective modifiers for asphalt [153,154].

The modification of asphalt with Styrene-Butadiene-Styrene (SBS) copolymer fundamentally enhances its viscoelastic character. It has been demonstrated that incorporating SBS broadens the glass transition temperature range of the base asphalt, which in turn significantly boosts elasticity and energy dissipation within critical service temperatures [155]. While the overall shapes of the viscoelastic curves (G′ and tan δ) for different SBS structures are consistent, their specific transition points and relaxation strength are directly influenced by the polymer’s composition. This link between structure and performance is key; comparative analysis has proven SBS-modified asphalt to possess superior damping properties over other polymers [156]. The root of this enhancement lies in the SBS molecule itself, with attributes like chain flexibility, steric hindrance, and elastic hysteresis being primary determinants of the final binder’s damping capacity. In fact, SBS and crumb rubber are recognized as the most effective polymers for this purpose. To further optimize these systems, the strategic use of additives is common. Crosslinkers, for instance, not only improve polymer compatibility and storage stability but also widen the effective damping temperature range [157]. Complementarily, plasticizers act by expanding the polymer network and increasing molecular motility, thereby further amplifying the material’s inherent energy dissipation mechanism [158].

### 5.2. Temperature

The behavior of asphalt is fundamentally governed by temperature, which directly controls the motion of molecules within the material. As temperatures rise, this molecular activity accelerates, allowing their relaxation processes to be observed over a much shorter timescale. A drop in temperature, conversely, slows these motions to a crawl. This intimate dance between heat and molecular movement dictates asphalt’s damping properties, leading to three distinct states: it becomes hard and brittle in the cold, transitions into a viscoelastic solid with pronounced damping behavior at moderate temperatures, and ultimately softens into a viscous fluid when heated. This temperature-dependent character is a hallmark of viscoelastic materials. Research has confirmed that in fiber-reinforced composites, a rise in temperature leads to a lower storage modulus and a higher damping loss factor [159]. Similarly, studies on asphalt materials have demonstrated that the effect of temperature on their damping properties aligns closely with the behavior observed in other viscoelastic damping systems [160].

The dynamic shear modulus (G) and loss factor (tan δ) of asphalt binders exhibit significant temperature-dependent viscoelastic behavior, typically characterized by four distinct rheological regions: the glassy state, the glass transition region, the rubbery plateau, and the terminal viscous flow region. Within these regions, the material’s damping properties vary substantially. The glass transition region is of particular functional importance, as it is marked by a precipitous decline in modulus concurrent with a rapid increase in the loss factor. This loss factor reaches a maximum—termed the damping peak—before undergoing a sharp decrease. Consequently, the glass transition region represents the primary domain in which asphalt binders express their damping capacity. This damping behavior is intrinsically governed by temperature, with optimal energy dissipation occurring within the glass transition range. Here, a pronounced material softening directly enhances the loss factor, signifying a peak in viscous energy absorption [161]. This phenomenon is not merely a macroscopic change in state but reflects a fundamental shift at the molecular level, where the internal structures begin to reconfigure [162]. A more comprehensive understanding of these damping mechanisms is achieved by examining the molecular motions within the asphalt, which become progressively more active and complex as temperature rises, as detailed in several supporting studies [163,164] and summarized in Table 3.

Consequently, the molecular fluidity increases as the temperature goes from low to high under constant external force. Microscopically, molecular motions contain abundant different forms. Macroscopically, the asphalt binders experience three states: glassy, highly elastic and viscous. The damping properties of asphalt binders vary in different environments, the glass transition zone is the characteristic working area of asphalt binders, where the asphalt binders exhibit the best damping properties when the glass transition temperature overlaps or approaches the ambient temperature.

### 5.3. Frequency

**Figure 8:**
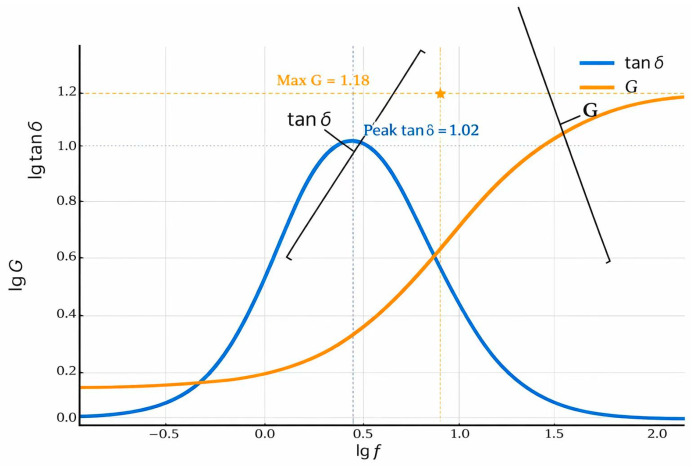
The curves of loss factor and modulus with frequency. The five-pointed star marks the point where the metric G reaches its maximum, with approximate coordinates (gf ≈ 1.0, G ≈ 1.18).

The energy dissipation capacity of viscoelastic materials exhibits a significant dependence on loading frequency, in addition to its well-known relationship with temperature [165,166,167]. Research has confirmed that both the elastic modulus and the damping loss factor (tan δ) are functions of frequency, underscoring the material’s time-dependent nature [168]. As illustrated in Figure 8, the loss factor demonstrates a non-monotonic relationship with frequency, varying distinctly across different material states. In the glassy region, tan δ decreases as frequency increases. Conversely, a rising trend is observed within the high elastic region. The most effective damping in asphalt binders is achieved at the peak of the tan δ curve within the glass transition zone, where the energy dissipation is maximized for a given frequency. The underlying mechanism for the decline in damping at elevated frequencies can be explained at the molecular level. Damping originates from the dislocation movements and micro-slip at material interfaces; however, under high-frequency alternating stress, the time for these motions to occur within a single cycle is reduced. This restriction leads to smaller displacements and consequently lower energy consumption per cycle, manifesting as poorer overall damping properties [169]. The corresponding evolution in the molecular motion states of asphalt binders across the frequency spectrum is detailed in Table 4. Ultimately, the condition for peak damping performance is the convergence of increasing temperature and decreasing frequency, which defines the glass transition zone where the loss factor reaches its maximum [170].

In short, frequency also affects the damping properties of asphalt binders. The higher temperature and lower vibration frequency, lower temperature and higher frequency provide the same effect on the damping properties of asphalt binders according to the principle of time-temperature equivalence. The strain of polymers involves only changes in bond length and bond angle, and the chain segment motions fail to keep up with the change in the external force, producing a small energy loss and the tan δ is almost 0 at the high frequency or low temperature. However, the motions of the chain segments keep up with the external force only at the appropriate temperature and frequency, but with a lag. tan δ behaves larger and the maximum occurs in the glass transition zone, the material exhibits optimal damping properties. Nevertheless, there are few studies on the damping properties of viscoelastic materials in different frequency ranges at present.

A comparative analysis of structural modifications for enhancing asphalt pavements is presented in Table 5. This synthesis systematically catalogues innovations at both the binder and mixture scales, evaluating the efficacy of specific additives and design paradigms. The table delineates the mechanistic role of various technologies—such as crumb rubber, SBS polymers, epoxy networks, and optimized porosity—in altering the material’s internal structure and improving its energy dissipation characteristics. Consequently, the resulting performance benefits, encompassing substantial noise reduction, improved durability, and superior mechanical strength, are detailed to furnish a clear evaluation of these advanced material technologies.

### 5.4. Comparative Analysis of Noise Mitigation Strategies: Mechanistic Damping vs. Structural Control

Although both mechanistic damping and structural control strategies seek to attenuate Tire-Pavement Interaction Noise (TPIN), their underlying operational principles are fundamentally distinct. These divergent mechanisms give rise to unique performance profiles, characterized by complementary advantages and inherent limitations. A comparative summary of these critical distinctions is presented in Table 6.

The comparative analysis presented in the preceding table delineates two fundamentally distinct paradigms for noise mitigation. The mechanistic damping strategy, governed by the intrinsic viscoelastic character of the asphalt binder, constitutes a material-centric solution. Its efficacy, derived from bulk property enhancement rather than surface characteristics, confers a superior resistance to performance degradation from superficial wear. A central challenge in its implementation, however, lies in navigating the inherent rheological trade-off: optimizing the loss factor (tan δ) for maximal energy dissipation must be balanced against maintaining a sufficient storage modulus (G) to ensure resistance to rutting and other forms of permanent deformation under traffic loading.

Conversely, structural control strategies are unequivocally geometry-centric, delivering potent noise abatement by directly targeting the generative mechanisms of TPIN, such as air pumping. Their principal limitation is one of durability; the functional porosity and engineered texture essential for acoustic absorption are intrinsically vulnerable to clogging from environmental detritus and mechanical degradation from traffic polishing. Consequently, these surfaces often exhibit a pronounced decay in acoustic performance over their service life, potentially necessitating interventions to restore their noise-mitigating function.

The selection of an appropriate strategy is therefore contingent upon project-specific priorities. The mechanistic approach provides a foundational, sustainable solution for long-term vibration damping, deeply integrated into the pavement matrix. In contrast, the structural approach offers a powerful tactical option where immediate and substantial noise reduction is the critical objective, albeit with an accepted risk of performance decline. For infrastructure demanding both immediate acoustic benefits and enduring performance, these strategies exhibit compelling complementarity.

Thus, the vanguard of quiet pavement design is increasingly characterized by a synergistic methodology. This integrated approach leverages a high-damping binder to address structure-borne vibrational noise at its source, while a meticulously engineered surface morphology concurrently mitigates aerodynamically generated noise. This dual-pathway strategy ensures a more resilient acoustic performance, safeguarding long-term noise reduction even as the efficacy of surface-based mechanisms may diminish.

## 6. Modern Computational Frameworks for Damping and Acoustic Performance

Recent advances in computational modeling have fundamentally enhanced the prediction and understanding of how the damping properties of asphalt mixtures govern their acoustic performance, heralding a shift towards a multi-scale and data-driven paradigm. This modern framework synergistically integrates high-fidelity finite element (FE) vibro-acoustic simulations, which employ first-order shear deformation theory and Rayleigh integrals to model transient acoustic pressure under dynamic loading, with established damping–noise correlation models that quantitatively link mixture composition to acoustic outcomes [171,172,173,174]. Furthermore, the field is increasingly leveraging machine-learning (ML) frameworks, including ensemble methods and neural networks, which utilize large experimental datasets to identify critical predictors—such as binder properties and gradation—and accurately forecast viscoelastic behavior and noise absorption [175,176,177,178]. The frontier of this research lies in multi-scale and hybrid modeling, which couples FE, discrete element method (DEM), and ML across scales to simulate the complex interplay between microstructure, damping mechanisms, and macroscopic acoustic performance, thereby enabling the virtual design of optimized, low-noise pavements [46,179,180,181].

## 7. Conclusions

This review systematically examines strategies for mitigating tire-pavement interaction noise (TPIN), establishing the viscoelastic damping capacity of pavement materials as a critical determinant of acoustic performance. This property governs the conversion of vibrational energy into heat, thereby reducing noise at its source. While macro-scale design parameters—such as surface texture and mixture porosity—can contribute to noise reduction, their efficacy is frequently constrained by compromises to long-term durability. Consequently, enhancing the intrinsic viscoelastic damping of paving materials emerges as a particularly viable and sustainable strategy for advanced TPIN abatement.

The promise of this material-centric approach is demonstrated through the modification of asphalt binders. The incorporation of polymer modifiers, inorganic fillers, or the application of epoxidation techniques can markedly improve damping properties. These enhancements are driven by microstructural mechanisms, such as improved modifier dispersion and the formation of cross-linked networks, which restrict molecular chain mobility and thereby augment energy dissipation. Critically, these performance gains are contingent on external conditions; temperature and loading frequency profoundly influence molecular dynamics and, consequently, the macroscopic damping behaviour.

Effective noise-reducing pavements therefore necessitate a holistic design that integrates these material-level insights with structural principles. This entails a synergistic optimization of material composition (crumb rubber, recycled constituents), structural configuration (layer thickness), and surface characteristics. Furthermore, the use of recycled materials and warm-mix technologies aligns acoustic objectives with environmental sustainability—a synergy increasingly supported by emerging Life Cycle Assessments that incorporate noise emission models.

In synthesizing these findings, this review underscores that the evolution of next-generation, low-noise pavements requires a coordinated, multi-scale research approach bridging material science, structural mechanics, and acoustics. A pivotal insight is the need for a paradigm shift in standards and specifications towards a performance-based framework that explicitly values and quantifies damping capacity, moving beyond a reliance on macro-texture alone. However, the translation of these insights into practice is currently hampered by significant research gaps, including the absence of unified damping measurement protocols and a pronounced laboratory-to-field validation gap.

To address these limitations, future work should prioritize several key directions:

1. The development of standardized, field-validated test methods to reliably correlate laboratory-measured damping properties with in-service acoustic performance.
2. The integration of advanced material models into numerical simulations of the complete tire-pavement system to improve predictive accuracy.
3. A deeper investigation into the evolution of surface texture and its acoustic implications throughout the pavement lifecycle.
4. The exploration of next-generation material systems—such as tailored nanocomposites, bio-based modifiers, and self-healing polymers—designed to synergistically enhance viscoelastic damping, structural resilience, and environmental sustainability.

Ultimately, the creation of quieter infrastructure will depend on a harmonized pursuit of acoustic performance, mechanical durability, and environmental responsibility.

## Figures and Tables

**Table 1:** Influences of Material Composition and Design on Pavement Damping and Noise Mitigation.

References	Material Composition	Layer Thickness	Surface Texture	Environmental Impact	Performance
[33,34,35,36,37,38,39,40,41,42,43,44,45]	Rubberized asphalt mixtures (especially crumb rubber) are highly effective for noise reduction, improved durability, and mechanical performance.	Optimized for specific mixture types (OGFC, dense mixtures) to balance noise absorption, durability, and structural integrity.	Texture, porosity, and sound absorption are critically linked to noise reduction and are intentionally designed through material selection and gradation.	Significant benefits from recycling waste tires, reducing emissions, and enhancing sustainability through extended service life.	Proven effective for urban traffic noise mitigation, with performance validated through laboratory and field tests under various conditions.
[46,47,48,49,50,51,52]	Mixtures (especially porous and two-layer) are designed to balance acoustic integrity (3+ dB noise reduction) with mechanical strength and skid resistance.	Thickness is a critical, optimized parameter for durability, acoustic absorption, and adaptation to urban speeds (~50 km/h).	Texture, porosity, and flow resistivity are tailored for noise mitigation, sound absorption, and skid resistance.	Often implicit through durability and material optimization; explicit benefits include rainwater management and recycling.	Validated for urban conditions (traffic loading, noise reduction) through lab, in situ, and full-scale road tests.
[53,54,55,56,57,58,59,60]	Void ratio, aggregate size, and mixture gradation are key factors for sound absorption and noise reduction.	Thickness is positively correlated with noise reduction and is optimized for pore resonance and medium void content.	Texture parameters (gradation, voids, depth, wavelength) critically define the noise absorption spectrum and generation mechanisms.	Largely unaddressed or only indirectly considered through material optimization and selection.	Primarily laboratory studies on acoustic properties, with some urban design recommendations and field evaluations.
[11,61,62,63,64]	Modified, recycled, and eco-friendly materials (RAP, PET, BIO-additives, warm mix) enhance noise reduction, durability, and mechanical performance.	Optimized or considered for durability, life cycle performance, and environmental benefits.	Contributes to noise reduction, permeability, and acoustic performance; effects are often implicit in overall analyses.	Comprehensive assessment shows benefits via recycled materials, reduced emissions, heat absorption, and exhaust decomposition.	Sustainable and applicable in urban settings with features for diverse climates, including anti-icing and long-term performance.
[12,13,65,66,67,68]	A range of materials (thin asphalt, porous asphalt, steel slag, crumb rubber SMA) provides effective initial noise reduction, with durability varying from 3–8+ years. Dense-graded mixes are less effective.	Thickness is typically optimized for a balance of noise reduction and mechanical durability, with thin layers being a common strategy.	Surface texture (macrotexture, clogging, degradation) is critically linked to noise evolution and performance deterioration over time.	Generally, not a primary focus, except for one case where recycling steel slag was noted to reduce environmental footprint.	Long-term performance (3–8+ years) is monitored under real-world urban and motorway conditions, showing variable durability and noise retention influenced by traffic and climate.
[69,70,71,72,73]	Aggregate, binder, and porosity are critical for acoustic performance, durability, and climate-specific suitability.	Thickness is optimized for noise reduction (urban/tunnels) and mechanical balance, often using predictive models.	Texture and void content are primary factors controlling noise generation, attenuation, and driver perception.	Largely implicit, considered through material sustainability and suitability for specific environments like cold climates.	Effective noise reduction is validated in diverse settings: urban streets, tunnels, motorways, and cold regions.

**Table 2:** Comparison of key studies on damping properties of pavement materials.

Reference	Material/Modification	Methodology	Key Results
[93]	Rubberized porous asphalt	Dynamic modulus, vibration-damping, CT imaging	Rubber content up to 3% improves damping and energy dissipation
[94]	SBS-modified bitumen	Loss factor spectra, molecular analysis	Wide damping temperature range, improved by crosslinker/plasticizer
[95]	Rubberized asphalt mixtures	Mix design, mechanical/damping tests	Higher damping than traditional mixes, good rut resistance
[96]	Epoxy asphalt rubber with WMA additive	Viscosity, glass transition, damping, morphology	Sasobit improves damping and workability, optimal at low concentrations
[97]	Damping asphalt mixtures (DAMs)	Mechanical, rutting, image analysis	High damping and rutting resistance as interlayer

**Table 3:** Variation in molecular motion state of asphalt binders with temperature.

Zones	State of Molecular Motion	Damping Properties
Glassy zone	The molecular chain fails to overcome the gravitational force between the molecular chains and convert the mechanical energy into thermal energy.	Asphalt binders exhibit almost no damping properties.
Glass transition zone	The strain lags behind the stress. The neighboring macromolecular chain and the various groups on the molecular chain move to produce internal friction, converting the mechanical energy to the greatest extent into the thermal energy.	Asphalt binders exhibit the best damping properties.
High-elastic zone	The molecular chain segment changes from the coiled coil state to the stretched state, having a great deformation but quickly recovers, failing to absorb enough mechanical energy.	The damping properties of asphalt binders are not the best.
Viscous-flow zone	The molecules offer almost no dynamic properties and the deformation failed to be restored.	Asphalt binders exhibit no damping properties.

**Table 4:** Variation on molecular motion state of asphalt binders with frequency.

Conditions	State of Molecular Motion	Damping Properties
High frequency	The frequency of external action is much greater than the inverse relaxation time of the chain segment motions. The molecular chain motions simply fail to keep up with the external forces, showing less internal friction.	Asphalt binders exhibit little damping properties.
Vitrification transition zone	The motions of the chain segments are in a semi-hysteresis state that fails to keep up with stress, also exhibiting a certain backwardness. The molecular chain motions reach the maximum.	Asphalt binders exhibit the best damping properties.
Low frequency	The chain segment motions completely keep up with the change in stress and the internal friction of the molecular chain segment is small.	Asphalt binders exhibit almost no damping properties.

**Table 5:** Mechanisms and performance outcomes of structural modifications in asphalt materials for enhanced damping and noise reduction.

Modification Strategy (Scale)	Fundamental Mechanism	Rheological & Functional Property Enhancement	Documented Performance Outcomes
Crumb Rubber (CR) Modification (Binder)	Dispersion of vulcanized rubber particles forming a resilient, cross-linked network that impedes crack propagation and dissipates energy.	- Marked increase in loss modulus (G) and loss factor (tan δ). - Broadened temperature range (ΔT) for effective damping. - Reduced thermal susceptibility of viscoelastic response.	- Field-validated traffic noise reduction ≥ 3 dB. - Superior resistance to rutting and fatigue cracking. - Sustainable valorization of end-of-life tires.
SBS Polymer Modification (Binder)	In situ formation of a three-dimensional swollen copolymer network, featuring rigid polystyrene (PS) domains for reinforcement and elastic polybutadiene (PB) domains for energy dissipation.	- Pronounced peak and plateau in tan δ within the service temperature range. - Enhanced elastic recovery and hysteresis. - A broadened glass transition region.	- Exceptional damping efficiency and noise abatement. - Improved flexibility and low-temperature performance. - High toughness and resistance to permanent deformation.
Epoxy Asphalt Formulation (Binder)	Irreversible covalent cross-linking creates a rigid thermosetting matrix that encapsulates the asphalt phase.	- Sustained high damping factor (tan δ) across a wide temperature spectrum. - Large integrated area under the tan δ curve (TA), denoting high total energy dissipation capacity.	- Outstanding long-term structural integrity and deformation resistance. - Effective damping of structural vibrations. - Indispensable for demanding applications (orthotropic steel bridge decks).
Warm Mix Additives with Modifiers (Binder)	Thermodynamic facilitation of modifier dispersion (CR, SBS), yielding a more homogeneous and continuous composite microstructure.	- Synergistic elevation of G and tan δ over the base modified binder. - Refined morphology reduces stress concentrations.	- Augmented damping performance from the primary modifier. - Enhanced practical workability, enabling reduced production temperatures and emissions.
Porous Friction Course Design (Mixture)	Implementation of a high-void (18–25%), interconnected pore structure that functions as a Helmholtz resonator and wave trap.	- Complementary sound energy dissipation via viscous losses and thermal effects within air voids. - Damping properties are primarily conferred by the modified binder matrix.	- Dual-mechanism noise attenuation: absorption + material damping. - Potential for ravelling and clogging necessitates use of robust, polymer- or rubber-modified binders.
Engineered Surface Texture (Mixture)	Strategic design of surface macrotexture to mitigate the generation mechanisms of tire-pavement interaction noise (TPIN).	- Not a material-dependent damping enhancement. - Source amplitude reduction via minimized impact forces, altered adhesion dynamics, and suppression of air-pumping resonance.	- Effective reduction in noise at the source. - Acoustic performance is highly morphology-specific and susceptible to deterioration from surface wear.

**Table 6:** Comparative Analysis of Pavement Noise Mitigation Strategies.

Feature	Mechanistic Damping (Binder-Level)	Structural Noise Control (Texture/Porosity)
Governing Principle	Material viscoelasticity; energy dissipation via internal friction.	Surface geometry; noise source reduction and acoustic absorption.
Primary Mechanism	Converts vibrational energy into heat at the tire-pavement interface.	Reduces air-pumping and impact; traps and attenuates sound waves in pores.
Targeted Noise	Low-frequency, structure-borne vibration noise.	Medium- to high-frequency aerodynamic and impact noise.
Performance Driver	Binder chemistry, polymer modifiers, temperature, loading frequency.	Surface macrotexture, porosity, pore size, and connectivity.
Key Advantage	Bulk material property; less susceptible to surface clogging or wear; integrated into pavement structure.	High initial noise reduction; effective for a broad frequency range.
Primary Limitation	Trade-off between damping efficacy and mechanical stiffness (rutting resistance).	Performance degradation over time due to clogging, polishing, and raveling.
Durability	High. Performance is tied to the material’s bulk aging, not surface wear.	Variable to Low. Highly dependent on traffic, environment, and maintenance; prone to fouling.
Optimal Application	Fundamental strategy for all noise-sensitive pavements; essential for durable, long-term noise reduction.	High-priority areas where immediate, significant noise reduction is critical; less critical for long-term performance.

## Data Availability

No new data were created or analyzed in this study. Data sharing is not applicable to this article.
